# Future fertility of patients with zero oocytes yield in their first IVF cycle attempt

**DOI:** 10.1371/journal.pone.0246889

**Published:** 2021-02-16

**Authors:** Raoul Orvieto, Jacob Farhi, Ravit Nahum, Shani Basch, Jigal Haas, Adva Aizer

**Affiliations:** 1 Department of Obstetrics and Gynecology, Chaim Sheba Medical Center (Tel Hashomer), Ramat Gan, Israel; 2 Sackler Faculty of Medicine, Tel Aviv University, Tel Aviv, Israel; 3 The Tarnesby-Tarnowski Chair for Family Planning and Fertility Regulation, at the Sackler Faculty of Medicine, Tel-Aviv University, Tel-Aviv, Israel; 4 IVF Unit, Wolfson Medical Center, Holon, Israel; Fondazione IRCCS Ca’ Granda Ospedale Maggiore Policlinico, ITALY

## Abstract

**Purpose:**

We aim to estimate the future fertility of patient undergoing their first IVF cycle attempt with no oocyte retrieved, and to identify factors that might predict those who will conceive in subsequent IVF cycle attempt.

**Methods:**

A cohort retrospective study of all consecutive women attending our IVF unit, for their first IVF cycle attempt, between January 2013 to December 2019, who reached the ovum pick-up (OPU) stage with zero oocyte retrieved. Patients’ characteristics and infertility-treatment-related variables in the first IVF cycle attempt were compared between those who conceived in a subsequent cycle and those who did not. Moreover, infertility-treatment-related variables during successful cycles resulting in pregnancy were compared to those without.

**Results:**

59 met the study inclusion criteria, yielding zero oocytes. During the follow-up period, 12 (20.3%) women conceived (one conceived twice), and 8 (14%) gave birth to a live infant. Cumulative live-birth rate per OPU and per patients were 4% and 14%, respectively. Clinical pregnancies were achieved after 3.61+1.4 cycle attempts (range: 1–6), with no live-births following the fifth IVF cycle attempt. No in-between group differences were observed in ovarian stimulation variables of their first IVF cycle attempt. Moreover, in those cycles resulting in pregnancy, patients achieved a significantly higher number of fertilized oocytes (2.15+1.5 vs 0.94+1.5, respectively; p<0.01) and a higher mean top-quality embryos (TQE) (1.76+0.9 vs 0.73+1.2, respectively; p<0.003).

**Conclusion:**

Women yielding zero oocytes at their first IVF cycle attempt, may achieve 14% cumulative live-birth rate after 5 IVF cycle attempts. Moreover, those who conceived in subsequent IVF cycle attempts were those achieving 2 or more fertilized oocytes/TQE.

## Introduction

Ovarian stimulation (OS) is one of the key factors for the success of in vitro fertilization (IVF), targeting for an optimal number of growing follicles that will produce competent oocytes. However, due to the extreme heterogeneity in ovarian response to OS, some patients may yield a limited number of follicles, if any [1]. The literature concerning women with no oocyte retrieved in their first IVF cycle attempt is somehow scarce. Moreover, most of studies *a priori* exclude patients with cycle cancellation or zero oocytes upon retrieval [2–4], with only few presenting their prevalence [5]. The reason probably lies in their inherent poor prognosis and their early referral to egg donation. While a figure of 4.7% (41 out of the 861) prevalence was reported in poor responder patients in the ESPART study [5], the prevalence in the general IVF population is obscure.

In Israel, the national health insurance provides financial coverage for repeated IVF treatments, with an almost unlimited number of cycles up to the age of 45 years, unless 3 consecutive cycles resulted with no embryos transferred. Since IVF is an intense and complex procedure associated with financial, physical and emotional burdens, it would be therefore extremely challenging to consult patients with zero oocytes retrieved in their first IVF cycle attempt, concerning their future reproductive outcomes, using their own eggs.

Prompted by the aforementioned observation, we aimed to estimate the future fertility of patients undergoing their first IVF cycle attempt with no oocyte retrieved, and to identify factors that might predict those who will conceive in subsequent IVF cycle attempt.

## Patient and methods

The present cohort retrospective study included all consecutive women attending our IVF unit, for their first IVF cycle attempt, between January 2013 to December 2019. Inclusion criteria were women who reached the ovum pick-up (OPU) stage with zero oocyte retrieved, that underwent at least one more IVF cycle attempt. The study was approved by the institutional review board, at Sheba medical center (SMC-20-7575). Consent was waived by the ethics committee (fully anonymized retrospective analysis).

Ovarian stimulation was initiated on the 3rd day of menses with the use of recombinant FSH (Gonal F, EMD Serono). Once the leading follicle reached a size of 13 mm, or E2 levels exceeded 1200 pmol/L, co-treatment with GnRH antagonist 0.25 mg/day (Cetrotide, Serono or Orgalutran, Merck) and recFSH+ recLH (Pergoveris, Serono) or highly purified human menopausal gonadotropin (Menopur, Ferring) were commenced. Follicle growth and hormone levels were serially monitored by ultrasound and blood tests. Triggering for final oocyte maturation was performed when the leading follicle reached 17–19 mm. Transvaginal oocyte retrieval was performed 36 h following trigger.

In the subsequent cycles, all the accepted protocols or medications for OS were included. The selection of type of OS protocol used was the decision of the treating physician and largely dependent on the fashion at the time. Clinical pregnancy was defined as visualization of a gestational sac and fetal heart activity on transvaginal ultrasound.

Data on patient age, infertility-treatment-related variables, laboratory and embryological data were collected from the patients’ medical files. Classification of embryo quality was based on previously published scoring parameters [6]; a top-quality embryo was defined as four to five blastomeres on day 2, seven or more blastomeres on day 3, equally-sized blastomeres and ≤10% fragmentation on day 3 and no multinucleation. The data from the first IVF cycle attempt with zero oocyte yield, as well as the outcome of their subsequent IVF cycles were recorded. Patients’ characteristics and infertility-treatment-related variables in the first IVF cycle attempt were compared between those who conceived in a subsequent cycle and those who did not. Moreover, infertility-treatment-related variables during successful cycles resulting in pregnancy were compared to those without.

Results are presented as means ± standard deviations. Normality of the data was tested using Shapiro-Wilk and Kolmogorov-Smirnov tests. Comparison of continuous variables between the two groups was conducted using Mann–Whitney U test. Significance was accepted at P < 0.05. Binary logistic regression was used for multivariate analysis. Statistical analyses were conducted using the IBM Statistical Package for the Social Sciences (IBM SPSS v.20; IBM Corporation Inc, Armonk, NY, USA).

## Results

During the study period, 3222 patients underwent 7589 IVF- OPU cycles, and 2876 underwent their first IVF cycle attempt, of whom 59 met the study inclusion criteria, yielding zero oocytes. Baseline clinical characteristics of the study participants at the commencement of first IVF cycle and OS variables at the first IVF cycle attempt are summarized in Table 1. Characteristics of the subsequent IVF cycles and patients’ final reproductive outcomes stratified according to IVF cycle rank are presented in Table 2. During the follow-up period, 12 (20.3%) women conceived (one conceived twice), and 8 (13.6%; 95% CI 7%-24.5%) gave birth to a live infant.

**Table 1:** Patients’ baseline clinical characteristics and cycle 1 OS variables.

Number of patients/cycles	59
Maternal age, years (mean ± SD)	38.7±4.7
Maternal BMI, Kg/m2 (mean ± SD)	26.66±7
Mean basal FSH IU/L (mean ± SD)	12.72±10.4
Mean basal LH IU/L (mean ± SD)	7.89±7.4
**Type of infertility**	
Male (%)	17%
Tubal (%)	5%
Endometriosis (%)	10%
Unexplained infertility (%)	47%
Ovulatory disorder (%)	5%
Uterine factor (%)	3%
Others (%)	12%
**OS characteristics**	
Duration of stimulation (days)	10.01±3.6
range	2–18
Total FSH dose used, IU (mean ± SD)	3783±2268
range	450–9000
Mean endometrium thickness, mms (mean ± SD)	9.03±2.6
range	4.7–20
Mean peak E2 pmol/L(mean ± SD)	1723±1710
range	186–8245
Mean peak P nmol/L (mean ± SD)	1.96±1.59
range	0.6–8.3

**Table 2:** IVF cycles’ characteristics and their final reproductive outcomes stratified according to IVF cycle rank.

IVF cycle rank	1	2	3	4	5	6	7	8	9	All
Duration of stimulation (days)	10.01+3.6	9.57+4.35	9.72+3.7	9.59+3.9	9.56+3.6	12.6+3.6	9.8+4	10.3+3	7	9.85+3.95
Total FSH dose used, IU (mean ± SD)	3783+2268	4378+2644	4570+2486	4813+2727	4951+2310	6307+2175	4470+2890	5450+2266	3150	4444+2554
Mean endometrium thickness, mms (mean ± SD)	9.03+2.6	8.75+2	9.04+2.1	8.77+2.04	8.56+1.88	9.42+1.1	8.84+1.2	10.33+0.4	9.2	8.82+2.2
Mean peak E2 pmol/L(mean ± SD)	1723+1710	2477+2334	2581+1694	2375+1677	1939+989	2470+1210	1205+265	2890+2125	4091	2210+1862
Mean peak P nmol/L (mean ± SD)	1.96+1.59	1.98+2.4	1.73+1	1.70+1.1	1.6+1.1	1.59+0.9	0.92+0.4	1.53+1.24	2.5	1.81+1.6
# of OPU with zero oocytes/total # of OPU	59/59	7/59	8/38	2/27	5/16	2/10	1/5	0/3	0/1	84/218
(%)	100%	12%	21%	7%	31%	20%	20%	0	0	
Total # of oocytes retrieved	0	127	108	69	37	21	6	10	7	385
Mean Oocytes/OPU (mean ± SD)	0	2.15+1.7	2.84+2.8	2.55+2.6	2.31+2.7	2.1+1.7	1.2+0.9	3.33+1.6	7	1.76+2.2
range	0	0–8	0–10	0–14	0–11	0–6	0–3	1–5	7	0–14
Number Fertilizes oocytes	0	65	65	42	27	7	3	6	6	221
Fertilization rate (%)	0%	51%	60%	61%	73%	33%	50%	60%	86%	57%
Mean fertilize/OPU (mean ± SD)	0	1.51+1.2	1.71+2.2	1.55+1.6	1.68+1.7	0.7+0.7	0.6+0.8	2+2.16	---	1.01+1.5
range	0	0–5	0–8	0–8	0–6	0–2	0–2	0–5	---	0
Number of TQE	0	55	42	38	22	7	3	5	2	174
TQE rate (%)	0	55/65	42/65	38/42	22/27	7/7	3/3	5/6	2/6	174/221
Mean TQE/OPU (mean ± SD)	0	0.93+0.9	1.1+1.5	1.4+1.6	1.37+1.3	0.7+0.7	0.6+0.8	1.66+1.6		0.79+1.2
Number of ET	0	34	17	18	10	5	2	2	1	89
Number of embryos transferred	0	47	30	30	18	7	3	4	1	140
Number of clinical pregnancy	0	5	0	4	3	1	0	0	0	13
Clinical Pregnancy rate/per transfer (%)	0%	15%	0	22%	19%	20%	0	0	0	15%
Number of sacs observed	0	5	0	6	3	1	0	0	0	15
Implantation rate (%)	0	11%	0	20%	17%	14%	0	0	0	11%
Cumulative clinical pregnancy/OPU	0	5/118	5/156	9/183	12/199	13/209	13/214	13/217	13/218	13/218
(%)	0%	4%	3%	5%	6%	6%	6%	6%	6%	6%
Cumulative clinical pregnancy/patient	0	5/59	5/59	9/59	12/59	13/59	13/59	13/59	13/59	13/59
Cumulative clinical pregnancy per patient rate (%)	0	8%	8%	15%	20%	22%	22%	22%	22%	22%
Number of live births	0	1	0	4	3	0	0	0	0	8
Live birth rate/ per transfer (%)	0	3%	0	22%	19%	0	0	0	0	8/59
Cumulative live birth/OPU	0	1/118	1/156	5/183	8/199	8/209	8/214	8/217	8/218	8/218
(%)	0%	1%	1%	3%	4%	4%	4%	4%	4%	4%
Cumulative live birth/patient	0	1/59	1/59	5/59	8/59	8/59	8/59	8/59	8/59	8/59
(%)	0	2%	2%	8%	14%	14%	14%	14%	14%	14%

**Fig 1:**
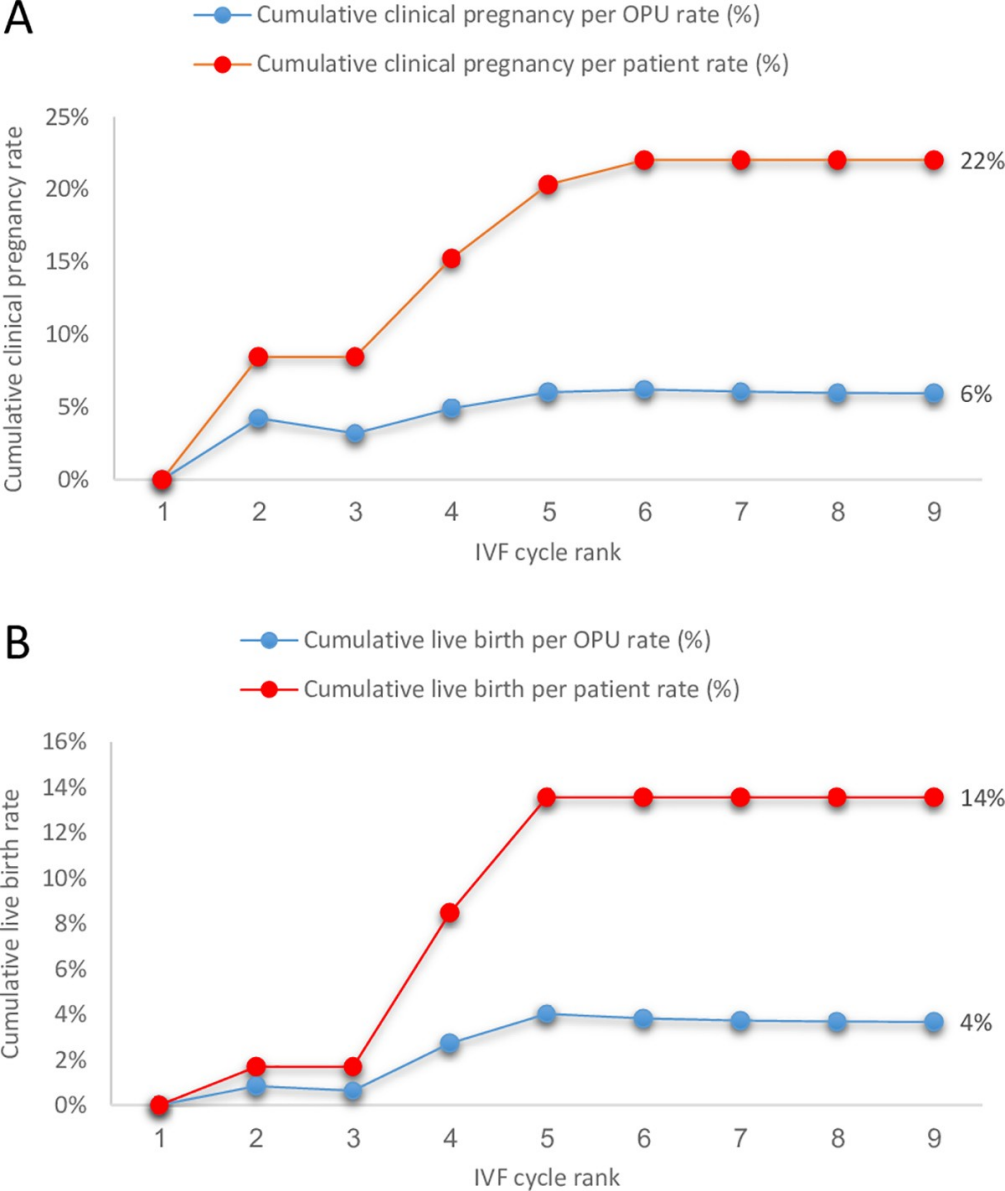
Cumulative reproductive outcomes stratified according to IVF cycle rank. **(a)** Cumulative pregnancy rate per OPU and per patient. **(b)** Cumulative live birth rate per OPU and per patient.

Mean implantation rate was 11%. Pregnancy and live-birth rates per transfer were 15% and 9%, respectively. Cumulative pregnancy rates per OPU and per patient were 6% and 22%, respectively (Table 2 and Fig 1). Moreover, cumulative live-birth rate per OPU (# of live births per total # of OPUs, up to the specific cycle rank) and per patients [# of live births in the cohort of patients who continued IVF attempts (59), up to the specific cycle rank] were 4% and 14%, respectively (Table 2 and Fig 1). Clinical pregnancies were achieved after 3.61+1.4 cycle attempts (range: 1–6), with no live-births following the fifth IVF cycle attempt.

A further analyses comparing women who achieved live birth versus those who did not are presented in Tables 3 and 4. No in-between group differences were observed in OS variables of their first IVF cycle attempt (Table 3). Moreover, while comparing the OS variables in the IVF cycle attempts resulting in clinical pregnancy vs those who did not, in those cycles resulting in pregnancy, patients achieved a significantly higher number of oocytes (3.23+2.5 vs 1.67+2.2, respectively; p< 0.002) and fertilized oocytes (2.15+1.5 vs 0.94+1.5, respectively; p<0.001) and a higher mean TQE (1.76+0.9 vs 0.73+1.2, respectively; p<0.001). No in-between groups differences were observed in the OS protocol or the type of gonadotropin medications used.

**Table 3:** OS variables in the first IVF cycle attempt of those who conceived vs those who did not.

	Preg	Non Preg	p-value
Number of patients	12	47	
Duration of stimulation (days)	10.66+3.1	10.06+3.9	0.51
Total FSH dose used, IU (mean ± SD)	3977+2397	3819+2284	0.82
Mean endometrium thickness, mms (mean ± SD)	9.64+4.1	8.88+2	0.85
Mean peak E2 pmol/L(mean ± SD)	1720+1041	1724+1842	0.24
Mean peak Progesterone nmol/L (mean ± SD)	2.22+2.4	1.74+1.2	0.75
Mean FSH on basal (mean ± SD)	12+6.1	10.5+4.9	0.49

**Table 4:** OS variables in the IVF cycle attempt resulting in clinical pregnancy vs those who did not.

	Preg	Non Preg	p-value
Number of treatment cycles	13	205	
Maternal age, years (mean ± SD)	39.61+3	39.08+4.6	0.95
Maternal BMI, Kg/m2 (mean ± SD)	21.35+13.7	27.24+7.4	0.86
Duration of stimulation (days)	9.38+3.47	9.89+3.9	0.68
Total FSH dose used, IU (mean ± SD)	4436+1907	4451+2591	0.87
Mean endometrium thickness, mms (mean ± SD)	8.75+1.5	8.93+2.2	0.58
Mean peak E2 pmol/L(mean ± SD)	2792+1858	2188+1864	0.24
Mean peak Progesterone nmol/L (mean ± SD)	1.35+0.6	1.76+1.6	0.5
Mean Cycles to reach pregnancy (range)	3.61+1.4 (2–6)	---	
Mean Oocytes/OPU (mean ± SD)	3.23+2.5	1.67+2.2	0.002
Mean fertilize oocytes/OPU (mean ± SD)	2.15+1.5	0.94+1.5	<0.001
Mean TQE/OPU (mean ± SD)	1.76+0.9	0.73+1.2	<0.001
Mean embryos transferred (mean ± SD)	1.69+0.9	1.55+0.7	0.66
Mean Stimulation days (mean ± SD)	9.38+3.47	9.89+3.9	0.68

Using a logistic regression model, controlling for age and BMI, we have found that TQE number significantly increased live birth rate (OR 1.55, 95%CI 1.07–2.23, p = 0.019).

## Discussion

The present study aims to provide women yielding zero oocytes at their first IVF cycle attempt, information regarding their chances of a live birth over their subsequent IVF treatment course and to examine whether parameters obtained during the first IVF cycle attempt may improve the predictive value.

The observed cumulative live-birth rate per OPU and per patient (4% and 14%, respectively) in the present study are in accordance with previous figures dealing with poor prognosis patients or those with diminished ovarian reserve [7, 8]. Moreover, while no parameters obtained during the first IVF cycle attempt in the current study could be of value in predicting patients’ future reproductive outcomes, patients who conceived in subsequent IVF cycle attempts following an attempt with zero oocytes, were those achieving 2 or more fertilized oocytes/TQE.

Previous studies have demonstrated that in women 40 years old and older undergoing their first IVF attempt, the only statistically significant differences between those who conceived and those who did not were patient’s age and the mean number of oocytes recovered [9]. Moreover, in agreement with our study, OS characteristics during the first IVF cycle attempt were unhelpful in predicting the possibility of clinical pregnancy within the first three consecutive IVF cycles.

Clinical pregnancies were achieved after 3.61+1.4 cycle attempts (range, 1–6), with no live births following the fifth attempt. We might therefore suggest that even when practicing in an environment with unlimited financial coverage, a patient should not undergo more than 5 IVF cycle attempts. This finding is in accordance with those of Lebovitz et al. [10]. While examining the cumulative incidence of live birth for high-order consecutive IVF cycles in women aged ≥41 years using autologous oocytes, they demonstrated that after six IVF cycles, most women delivered a live infant (85.7% of the total live birth). Again, multivariable regression models identified patient’s age and mean number of fertilized oocytes as factors significantly associated with the probability of a live birth [10].

IVF treatment involves high financial costs which significantly hinders the number of cycles that can be carried out. Yet, for countries in which partial or limited financial coverage for IVF treatments are available, the findings of the present study suggest the efficacy of extending the number of IVF cycles (for women with zero oocytes yield in their first IVF attempt) up to the fifth cycle, as most live births will occur within the first five IVF cycle attempts.

The limitations of this study are the relatively small number of participants included and its retrospective nature, which limits the ability to control for potential unknown confounding factors.

In conclusion, women yielding zero oocytes at their first IVF cycle attempt, may achieve 14% cumulative live-birth rate after 5 IVF cycle attempts. Moreover, those who conceived in subsequent IVF cycle attempts were those achieving 2 or more fertilized oocytes/TQE.
